# Cryptococcus Meningitis and reversible hearing loss

**DOI:** 10.1016/S1808-8694(15)31055-7

**Published:** 2015-10-19

**Authors:** Janini Oliveira Matos, Andréia Migueres Arruda, Shiro Tomita, Patricia de Pinho Marques Araujo, Felipe Barbosa Madeira, Krishnamurti Matos de Araujo Sarmento Junior

**Affiliations:** 1 Second-year resident of otolaryngology.; 2 Third-year resident of otolaryngology.; 3 Full Professor of Otolaryngology, Head of the Otolaryngology Service.; 4 Third-year resident of otolaryngology.; 5 Otolaryngologist.; 6 Otolaryngologist. Per-oral Endoscopist. Otorhinolaryngology Department - Clementino Fraga Filho University Hospital – Federal University of Rio de Janeiro.

**Keywords:** cryptococcus meningitis, reversible hearing loss

## INTRODUCTION

Cryptococcosis is the most common fungal infection to strike the Central Nervous System. Its sub-acute manifestation includes fever, headache, nausea, and changes in behavior. Dysacousia takes place in up to 27% of patients, almost always bilaterally and suddenly^1^. Reversibility is rare and was described by Mayer et al. in 1990. In general terms, if no treatment is offered death is the ultimate consequence.

## CASE STUDY

AJGG, female, 29 years of age, arrived in our center on December 25, 2003 with intense headache, discomfort, nausea, diplopia and reduced visual acuity; the symptoms had begun manifesting themselves two months before.

Her neck was stiff and the right abducens paretic. CBC came back normal and the quick HIV test was negative. Latex test, nankin dye and LCR indicated Cryptococcosis. CT scan revealed cerebral edema.

The patient evolved to hypoacousia and then quickly to blindness and bilateral total hearing loss. The otolaryngological evaluation was normal, except for the paretic abducens.

The patient’s condition made it impossible for audiometric tests to be conducted. BERA had thresholds at 110 dBSPL to the right and 100 dBSPL to the left, compatible with severe bilateral hearing loss for clicks. It was not possible to collect duplicated responses at 130 dBNPL and thus waves I, III and V were not analyzed. Otoacoustic emissions were normal.

**Figure:**
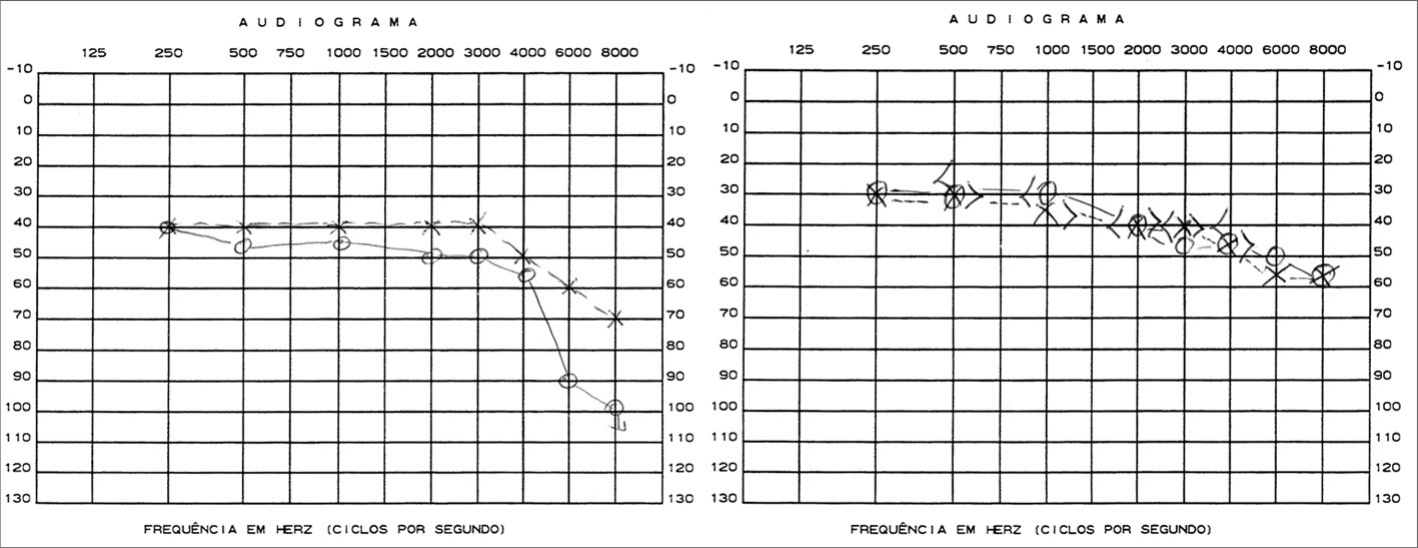

Treatment was initiated with intravenous Amphotericin B 50mg/day, getting to a dose of 1725 mg. The patient required a number of relief spinal taps and a ventriculoperitoneal shunt.

After 100 days in the hospital the patient improved clinically and was discharged without headache and neck stiffness, with partially recovered auditory acuity (picture) and multiple negative LCR cultures for fungi.

## DISCUSSION

Cryptococcal meningitis may occur at any given age and is more prevalent in immunologically compromised patients ^1^.

Hypoacousia is described in as many as 27% of patients and may fluctuate^1^, with losses ranging from moderate unilateral to severe bilateral^2^. The mechanism leading to lesions in the auditory system has been studied. Igarashi et al.^3^ and Kwartler et al.^4^ have seen destruction of cochlear and vestibular structures, with the presence of microorganisms in the vestibulocochlear nerve, internal auditory meatus and cochlear structures.

Harada et al.^5^ have seen most of these alterations, however with normal Corti’s organ and preserved vestibular nerve in relation to the cochlear nerve. Therefore, despite the apparent disagreement as to the involvement of the cochlea, all authors concur that there is retrocochlear damage^2^. In this sense, normal otoacoustic emissions suggest preservation of the Corti’s organ.

Mayer et al. described Hypoacousia reversibility in 1990^8^. The mechanism is however not fully comprehended.

Treatment of HIV-negative patients is done with amphotericin B and/or flucytosine for 6-10 weeks. This is a severe, potentially deadly disease.

## CONCLUSIONS

Cryptococcal meningitis is a severe infection and its clinical signs are of difficult diagnosis. Auditory involvement seems frequent, and its reversibility, rare. Early treatment is therefore of utmost importance, as is audiometric monitoring in the cases being followed.

## References

[1] Hughes KV, Green JD, Alvarez S, Reimer R (1997). Vestibular dysfunction due to cryptococcal meningitis. Otolaryngol Head Neck Surg.

[2] Low WK (2002). Cryptococcal meningitis: implications for the otologist. ORL J Otorhinolaryngol Relat Spec.

[3] Igarashi M, Weber SC, Alford BR, Coats AC, Jerger J (1975). Temporal bone findings in cryptococcal meningitis. Arch Otolaryngol.

[4] Kwartler JA, Linthicum FH, Jahn AF, Hawke M (1991). Sudden hearing loss due to AIDS-related cryptococcal meningitis-a temporal bone study. Otolaryngol Head Neck Surg.

[5] Harada T, Sando I, Myers EN (1979). Temporal bone histopathology in deafness due to cryptococcal meningitis. Ann Otol Rhinol Laryngol.

